# Resistance to Cypermethrin Is Widespread in Cattle Ticks (*Rhipicephalus microplus*) in the Province of Punjab, Pakistan: In Vitro Diagnosis of Acaricide Resistance

**DOI:** 10.3390/pathogens11111293

**Published:** 2022-11-04

**Authors:** Zia ud Din Sindhu, Muhammad Usman Naseer, Ali Raza, Bilal Aslam, Javed Ahmad, Rao Zahid Abbas, Muhammad Kasib Khan, Muhammad Imran, Muhammad Arif Zafar, Baharullah Khattak

**Affiliations:** 1 Department of Parasitology, University of Agriculture Faisalabad, Faisalabad 38000, Pakistan; 2 Queensland Alliance for Agriculture and Food Innovations, The University of Queensland, Brisbane 4072, Australia; 3 Institute of Physiology and Pharmacology, University of Agriculture Faisalabad, Faisalabad 38000, Pakistan; 4 Livestock Production Research Institute, Bahadurnagar, Okara 56300, Pakistan; 5 Department of Clinical Studies, PMAS Arid Agriculture University Rawalpindi, Rawalpindi 46300, Pakistan; 6 Department of Microbiology, Kohat University of Science and Technology, Kohat 26000, Pakistan

**Keywords:** cattle tick, cypermethrin resistance, larval packet test, resistance factor

## Abstract

Control of the cattle tick *Rhipicephalus* (*R.*) *microplus* mainly relies on chemical acaricides and cypermethrin is the most widely used acaricide in Pakistan. Farmers frequently complain about its low efficacy, thus, the present study was designed to quantify the frequency of cypermethrin resistance in cattle ticks. Engorged female *R. microplus* were collected and tested for the efficacy of cypermethrin using the FAO-recommended larval packet test. Resistance factors (RF) were estimated at both the lethal concentration for 50% (LC_50_) and 99% (LC_99_) of ticks. Thirty-three samples were tested, of which 8/33 (24.24%) were classified as resistant based on the RF_50_, and all 33 were classified as resistant based on the RF_99_. In District Sargodha, when only the RF_50_ was considered, 45.5% of samples were classified as resistant, but at RF_99_, all tested samples were identified as resistant. In District Okara, the variation in RF_50_ estimates was 2.2–8.3 and variation in RF_99_ estimates was 10.6–1139.8. Similar results were found in District Attock, where variations in RF_50_ were 0.8–8.5 and RF_99_ ranged from 9–237.3. The study showed that cypermethrin resistance is prevalent in these three districts of Pakistan and is likely to be overestimated by classification based on the RF_99_.

## 1. Introduction

Different types of parasitic infections are the major cause of reduced productivity and economic losses to livestock producers [1,2,3]. Among other parasites, infestation with ticks is one of the most important impediments to profitable livestock farming. Ticks are obligate blood-sucking parasites that infest a wide range of hosts including humans and have a worldwide distribution. They transmit most of the diseases as vectors, only second to mosquitoes [4,5]. Ixodidae is the dominant family of ticks regarding the number of species and their medical and veterinary significance [4,6]. They parasitize a huge range of hosts and are considered to be close to mosquitoes in their capacity to transmit pathogens such as bacteria, viruses, protozoa, and rickettsia to humans, domestic animals, and livestock [7]. In the case of livestock enterprises, 80% of the world’s cattle population is affected by ticks and tick-borne diseases [8].

Cattle tick *Rhipicephalus* (*R.*) *microplus* is considered to be of great significance [9,10] and it is distributed in tropical and subtropical regions of the world including Central and South America, Australia, India, China, and Malaysia [11]. The one-host tick *R. microplus* is the most important tick species from an economic point of view with cattle as a primary host. Severe losses are caused by this species through blood loss, tick worry, hide damage, production of toxins, and transmission of tick-borne diseases. Each engorged *R. microplus* causes a reduction in weight gain of just over 1.0 g during its repletion [12].

Control of ticks mainly relies on chemical acaricides [13] but many *R. microplus* populations have become resistant to most commercially available acaricides [14,15,16]. The synthetic pyrethroid (SP) class of organic insecticides was originally derived from natural pyrethrins. They have been used for tick control since the 1980s due to their greater effectiveness and lower mammalian toxicity as compared to other insecticides such as carbamic esters and organophosphorus compounds [17]. Pyrethroid pesticides are in the top three major pesticides globally [18] and the value of the synthetic pyrethroid market was $1.6 billion in 2016 [19]. Cypermethrin, deltamethrin, permethrin, bifenthrin, cyfluthrin, λ-cyhalothrin and fenvalerate are all widely used [20].

The larval packet test (LPT) developed by Stone and Haydock [21] is recommended by the Food and Agricultural Organization (FAO) as an effective bioassay to quantify acaricide resistance in ticks and to assess the candidate acaricides. The prevalence of tick infestation in cattle exceeds 50% in Pakistan, but very little information is available on the emergence of acaricide resistance [22], where no study has been conducted using FAO’s recommended LPT. Results based on LPT may give a better estimate of acaricide resistance as these results can be compared with international data for a better understanding of the situation. Thus, the present study was designed for the first time in Pakistan to quantify the prevalence of cypermethrin resistance among cattle ticks collected from three districts located in different agro-climatic regions of Punjab, Pakistan by using the LPT.

## 2. Materials and Methods

Three different districts (administrative units), Sargodha, Okara, and Attock, located in different agro-climate regions of the Punjab Province were selected for this study. District Sargodha is located at 32.1566° N to 72.8043° E, which is an agricultural district (5864 km^2^) with wheat, rice, citrus trees, and sugarcane as its primary agricultural products. The community comprises flat, fertile plains with low hills. The river Jehlum flows on the western and northern sides, and the river Chenab lies on the eastern side of Sargodha. The riverine areas are used as pasture for grazing livestock. The mean precipitation is 189 mm, and temperature varies from 5–23 °C during winter to 25–49 °C in summer. District Okara is located at 30.801380° N to 73.448334° E and shares its border with India in the south-east, and other sides of the district are surrounded by neighborhoods of Bahawalnagar (south), Sahiwal (west) and Faisalabad (north). It has plain agricultural land, and the district’s total area is about 4377 km^2^, which is irrigated by the river Ravi running on the north border of the district. The annual average rainfall in Okara District is 548 mm. Attock District has a climate of hot summers and cold winters; the maximum temperature reaches 40 °C. Geographically, the community is mainly of hills, plateaus, and dissected plains located at 33°46′20 N and 72°22′6 E. It has an altitude of 348 m, and the average annual rainfall in the district is 783 mm.

Collection of ticks: A total of 33 livestock farms (both government and private) were visited for the collection of tick samples. At each farm, samples of 10–50 engorged female *R. microplus* ticks were collected by hand. At least 10 farms were selected from each district. The ticks were placed in ventilated, escape-proof plastic containers with a few blades of green grass to maintain moisture. The samples were kept cool and transported to the University of Agriculture, Faisalabad’s laboratory for further processing.

Larval Packet Test (LPT): The LPT was performed according to the protocol of FAO [23]. After washing and drying the engorged female ticks, they were incubated at 27–28 °C with 85–90% relative humidity (RH) for laying eggs. Eggs were collected on day 14 and transferred into individual tubes and then again incubated at the same conditions of temperature and RH for the hatching. Fourteen day-old larvae were used in LPT for diagnosis of resistance.

Acaricides: Technical grade 89% pure cypermethrin was used to prepare the stock solution in trichloroethylene: olive oil (2:1). For experimental bioassays, 50% serial dilutions (3.2, 1.6, 0.8, 0.4, 0.2 and 0.1%) of acaricide were prepared from the stock solution and tested against *R. microplus* collected from each of the farms.

Preparation of filter paper packets: A volume of 0.67 mL of each test solution was applied to a 7.5 × 8.5 cm piece of filter paper (control/treated), which was folded in half horizontally, and a single bulldog clip was slid up against each short side of the paper. A small cluster containing approximately 100 larvae was packed in the packet and closed with bulldog clips. The closed packets were laid on a tray for incubation at 27–28 °C and 85–90% RH for 24 h. After treatment, larvae that were capable of moving were considered to be alive. All other larvae, including those that moved their appendages but did not walk, were counted as dead.

Calculation of resistance factor: LPT mortality results were evaluated using the Probit (dose–response) analysis in Polo-Plus software [24]. The RF_50_ was obtained using the LC_50_ value of the test sample divided by the LC_50_ value for susceptible ticks. The Media Joya (CENAPA) cypermethrin-susceptible strain (LC_50_ = 0.013 and LC_99_ = 0.046) was used as a reference strain because no reference was available for Pakistan. The Media Joya reference values are recommended by the FAO [25]. The RF_99_ was derived similarly. To classify the level of susceptibility of *R. microplus* to cypermethrin, the following criteria were used: RF < 3: susceptible; RF = 3–5: tolerant; and RF > 5: resistant [26]. The Chi-squared test was used to discriminate susceptible from resistant samples, considering both RF_50_ and RF_99_.

## 3. Results

**Figure 1:**
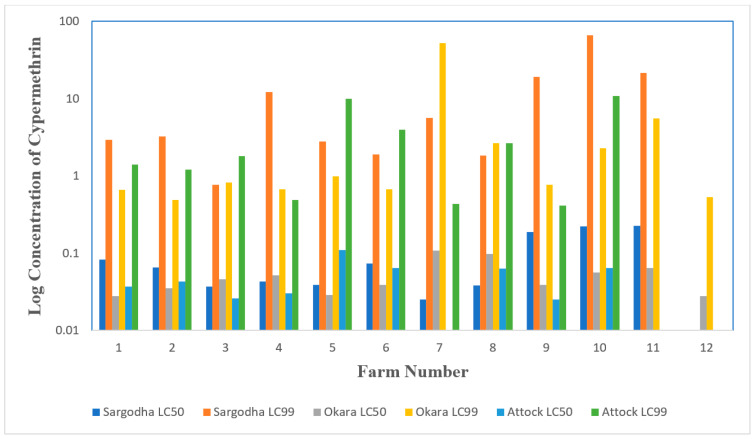
Bar diagram showing Cypermethrin lethal concentration at different livestock farms in three selected districts.

Engorged female *R. microplus* ticks collected from District Sargodha started to lay eggs after 12 days of incubation and continued until day 17. Results of LPT conducted at ticks collected from District Sargodha are presented in Table 1 and Figure 1. A wide variation was found in the RFs of the samples from each of the farms (RF_50_: 1.92–17.46; RF_99_: 16.76–470.97). The RF_99_ suggested that all samples were resistant. When the level of resistance was determined using the RF_50,_ 45.5% of all samples were classified as resistant, while susceptible and tolerant samples were 27.3% each (Table 1).

In District Okara, variation in the RF_50_ was not as broad 2.2–8.3, (Table 2; Figure 1), however, a wide variation was found in RF_99_ in the samples tested (RF_99_: 10.6–1139.8). With RF_50_, a total of 16.7% resistant samples were found, while susceptible and tolerant samples were 33.3% and 50%, respectively. Similarly, in District Attock, variation in RF_50_ was also close (0.8–8.5), however, a wide variation was found in RF_99_ in the samples (RF_99_: 9–237.3). When the level of resistance was determined using the RF_50_, 10% of the samples were resistant, while susceptible and tolerant samples comprised 50% and 40%, respectively (Table 3; Figure 1).

## 4. Discussion

The cattle tick *R. microplus* is one of the major ectoparasites infesting cattle and causing huge economic losses to the cattle industry worldwide. The cattle tick has become resistant to many classes of acaricides including pyrethroids [27]. Many reports about the development of acaricide resistance have been documented in South East Asia, Australia, the Caribbean, and Central America (reviewed by [28]). Classification of acaricide-resistant, -tolerant and -susceptible strains based on dose–response bioassays is challenging, regardless of the precision of the bioassays. The ideal approach is to conduct identical binary quantal response bioassays in test and susceptible reference samples, with resistance being defined as non-overlapping confidence intervals in the slope of the response [29]. However, susceptible reference samples can be challenging to obtain and the application of resistance factors (RF = LC test sample/LC susceptible reference sample) in which the susceptible LC is derived from a well-documented susceptible reference strain is an alternative. In the present study, the confidence intervals for RF were much wider for the estimates based on LC_99_ than estimates based on LC_50_. The fact that LC_50_ is statistically more powerful than LC_99_ is expected in any bioassay. The estimation of RF_99_ from LC_99_ values further compounds the error associated with the estimation of LC_99_ from each of the resistant and susceptible samples. It is, therefore, not surprising that the confidence intervals in the estimates of RF_99_ were consistently much higher than the estimates of RF_50_ in the present study. The lack of precision of estimates of LC_99_ has been noted previously for LPT on cattle ticks [30] and the subject of bioassay design and statistical analysis is extensively reviewed elsewhere [29]. Given the limitations of RF_99_ in general and the wide confidence intervals we found, we consider that the classification of phenotypes should be based on the RF_50_ estimates.

This study revealed a high overall prevalence (24%) of resistance to cypermethrin in the three selected districts of Pakistan, using estimates based on the RF_50_. Wide variation was found in RFs of the ticks collected from District Sargodha. There was less variation in the RF_50_ estimates of the other two districts (Okara and Attock). This can be associated with factors such as the migration of ticks among farms, animal breed, favorable environment for non-parasitic phase survivability, presence or absence of refugia, and the intensity of acaricide used [31]. Kunz and Kemp [32] reported that the development and level of resistance to ixodicides among tick populations depend on the frequency of resistant individuals in the population and the intensity of selection pressure. The severity of resistance in ticks has reached a stage where it is expected that ticks will be resistant to acaricides after 5–10 years of their introduction, and *R. microplus* is the tick species with the most studies regarding acaricide resistance [33].

Acaricide resistance arises through distinct molecular mechanisms, for example, metabolic/ detoxification enzymes, target-site modification, or reduced penetration [34]. Synthetic pyrethroids have the most widespread resistance and the highest resistance factors (>100) compared with other ectoparasiticides worldwide [35]. The previously documented mechanisms of pyrethroid resistance in *R. microplus* populations include detoxification enzymes such as monooxygenases [36] and esterases [37]. Target site insensitivity confers the highest resistance factors and is a result of mutations in the *para*-sodium channel [38]. None of the RF_50_ estimates in the present study exceeded 20. In contrast, Lovis et al. [39], using the larval tarsal test, found RF_50_ values from populations of *R. microplus* ranging from 1.7 to about 310 (with one apparent outlier > 2300) in Brazil, Argentina, Mexico, South Africa, and Australia.

## 5. Conclusion

This study showed that cypermethrin resistance is prevalent in all three selected districts. The status of acaricide resistance may be severe in Pakistan, which can be associated with no acaricide rotation policy, weak surveillance programs of resistance to acaricides, indiscriminate selling of acaricides, and no training about the rational use of acaricides. Such practices promote selection pressure and it will be difficult to control acaricide resistance once established. Thus, it is critical to acquire the status of acaricidal resistance at the national level. In conclusion, effective tick management should be guided by conducting nationwide surveillance programs and the periodic investigation of the development of acaricide resistance in Pakistan.

## Figures and Tables

**Table 1:** Cypermethrin lethal concentration (LC), resistance factor (RF), slope, and phenotype derived from dose–response larval packet test bioassays of *Rhipicephalus microplus* collected from different livestock farms in District Sargodha.

Farm No.	50% Mortality			99% Mortality			Slope	Phenotype	
	LC_50_	95% Cl	RF	LC_99_	95% Cl	RF		RF_50_	RF_99_
1	0.082	0.046–0.120	6.30	2.951	1.521–9.301	64.15	1.494	R	R
2	0.065	0.028–0.106	5	3.258	1.430–17.284	70.82	1.367	T	R
3	0.037	0.007–0.067	2.84	0.771	0.349–10.871	16.76	1.768	S	R
4	0.043	0.023–0.065	3.30	12.080	5.699–38.329	262.60	0.950	T	R
5	0.039	0.018–0.063	3	2.791	1.399–9.414	60.67	1.254	T	R
6	0.073	0.028–0.121	5.61	1.901	0.844–12.877	41.32	1.640	R	R
7	0.025	0.008–0.048	1.92	5.666	2.282–33.258	123.17	0.989	S	R
8	0.038	0.023–0.054	2.92	1.834	1.119–3.881	39.86	1.380	S	R
9	0.187	0.135–0.246	14.38	19.099	10.154–46.399	415.19	1.159	R	R
10	0.221	0.152–0.302	17	65.882	27.119–246.107	1432.21	0.940	R	R
11	0.227	0.155–0.316	17.46	21.665	8.776–96.866	470.97	1.176	R	R

CI = Confidence interval.

**Table 2:** Cypermethrin lethal concentrations (LC), resistance factor (RF), slope, and phenotype derived from dose–response larval packet test bioassays of *Rhipicephalus microplus* collected from livestock farms in District Okara.

Farm No.	50% Mortality			99% Mortality			Slope	Phenotype	
	LC_50_	95% Cl	RF	LC_99_	95% CI	RF		RF_50_	RF_99_
1	0.028	0.016–0.041	2.2	0.662	0.428–1.368	14.4	1.698	S	R
2	0.035	0.017–0.051	2.7	0.487	0.298–1.325	10.6	2.036	S	R
3	0.046	0.031–0.059	3.5	0.816	0.550–1.487	17.7	1.857	T	R
4	0.052	0.039–0.065	4.0	0.672	0.471–1.143	14.6	2.101	T	R
5	0.029	0.008–0.053	2.2	0.987	0.478–5.761	21.5	1.524	S	R
6	0.039	0.015–0.061	3.0	0.673	0.355–3.227	14.6	1.883	T	R
7	0.108	0.065–0.157	8.3	52.433	20.233–228.863	1139.8	0.866	R	R
8	0.097	0.024–0.184	7.5	2.633	0.943–71.823	57.2	1.624	R	R
9	0.039	0.010–0.067	3.0	0.766	0.366–6.623	16.7	1.804	T	R
10	0.056	0.012–0.107	4.3	2.292	0.840–46.045	49.8	1.445	T	R
11	0.064	0.014–0.128	4.9	5.585	1.767–129.372	121.4	1.199	T	R
12	0.028	0.011–0.044	2.2	0.534	0.312–1.704	11.6	1.824	S	R

CI = Confidence interval.

**Table 3:** Cypermethrin lethal concentrations (LC), resistance factor (RF), slope, and phenotype derived from dose–response larval packet test bioassays of *Rhipicephalus microplus* collected from different livestock farms in District Attock.

Farm No.	50% Mortality			99% Mortality			Slope	Phenotype	
	LC_50_	95% Cl	RF	LC_99_	95% Cl	RF		RF_50_	RF_99_
1	0.037	0.014–0.061	2.8	1.414	0.705–5.919	30.7	1.469	S	R
2	0.043	0.023–0.064	3.3	1.206	0.684–3.323	26.2	1.609	T	R
3	0.026	0.003–0.057	2.0	1.807	0.694–30.530	39.3	1.265	S	R
4	0.030	0.017–0.041	2.3	0.489	0.327–0.970	10.6	1.911	S	R
5	0.110	0.063–0.163	8.5	9.918	4.42–38.534	215.6	1.190	R	R
6	0.064	0.030–0.103	4.9	3.980	1.750–19.453	86.5	1.299	T	R
7	0.010	0.002–0.021	0.8	0.436	0.264–1.278	9.5	1.421	S	R
8	0.063	0.036–0.092	4.8	2.635	1.408–7.554	57.3	1.433	T	R
9	0.025	0.013–0.037	1.9	0.416	0.276–0.876	9.0	1.913	S	R
10	0.064	0.022–0.114	4.9	10.914	3.423–144.751	237.3	1.043	T	R

CI = Confidence interval.

## Data Availability

Not applicable.
